# Meeting of the American Dental Associations

**Published:** 1923-09

**Authors:** 


### DENTAL REGISTER

THE MAGAZINE OF DENTISTRY

Vol. LXXVII SEPTEMBER, 1923 No. 9

### MEETING OF THE AMERICAN DENTAL ASSOCIATIONS.

### Program of the Scientific Sections

### Operative Dentistry, Materia Medica and Therapeutics

(Auditorium at Cleveland Hotel. All subsequent meetings held in same place.)

---

Officers of Section—J. R. Blayney, Chairman, Chicago, Ill.; E. C. Mills, Vice-Chairman, Columbus, Ohio; Alfred P. Lee, Secretary, Philadelphia, Pa.

Tuesday Afternoon, September 11th
2:00 P. M. to 5:00 P. M.

"Children's Teeth with Special Reference to Cavity Preparation and Choice of Filling Materials," by William H. Leak, New York, N. Y.

Synopsis—Prevention of decay. Method for parent to use in brushing the child's teeth. How the child should brush the teeth. Warning on the mal-use or misuse of prophylactic filling materials or of medication. Anatomical differences in permanent and deciduous teeth and their significance in cavity preparations and the choice of filling materials. When to save deciduous non-vital teeth. The importance of careful technic and its appreciation by the parent.

Discussed by Haidee Weeks Guthrie, New Orleans, La.; Philip R. Thomas, Minneapolis, Minn.

"Important Points the General Practitioner Should Know in Order to Prevent Dental Irregularities," by Mildred W. Dickerson, Washington, D. C.

Synopsis—A resume of what constitutes dental irregularities. Importance of careful and thorough diagnosis of mouth conditions early in childhood, including a history of inherited tendencies and predispositions of the child, that the more obscure irregularities may not be overlooked. Necessity for the unceasing watchfulness and care by the dentist during the periods of babyhood, childhood and adolescence. Orthodontia when indicated or when contraindicated. A plea for more uniform and simple instructions for home care of children's mouths.

Discussed by C. N. Johnson, Chicago, Ill.; Russell W. Bunting, Ann Arbor, Mich.

WEDNESDAY MORNING, SEPTEMBER 12th
9:00 A. M. to 12:00 M.

"The Densist's Chance of a Lifetime," by H. L. Rockwood, M. D., Cleveland, Ohio.

Synopsis—Why play a losing game when there is a way to win? The chances for good health and long life today depend more than ever before on how you play the game. Some rules of the game the dentist should know. The physical welfare of the dentist concerns the nation. Factors which conserve human life must themselves be conserved. Community responsibility. Individual responsibility. When in doubt play trumps. What are the trump cards in playing for health?

Discussed by Thomas P. Hinman, Atlanta, Ga.

"Gold Foil," by Edmund Noyes, Chicago, Ill.

Synopsis—The paper will give a brief account of the use of gold foil in dentistry, of noncohesive and cohesive gold, and the work of Dr. G. V. Black on that subject. The prevalence of imperfect operations as related to the widespread acceptance of the "New Departure" theories. Something about instruments and technic, also the relative uses for foil fillings and gold inlays.

Discussed by W. R. Clack, Mason City, Iowa; Willis A. Coston, Topeka, Kansas.

"Technic for Making Cast Gold Inlays," by Victor T. Nylander, Chicago, Ill.

Synopsis—A. Finishing of cavity preparation. Cavity protection. Waxes, wax pattern. B. Contour and contact.

C. Investment. Spatulation, application and setting. D. Dissipation of wax, rapid and slow method, temperature range. E. Blowpipes and casting: Oxygen gas; oxygen and hydrogen; hydrogen and air; hydrogen, gas and air. F. Golds. G. Finishing. H. Setting.

Discussed by Alden J. Bush, Columbus, Ohio; Robert O. Green, St. Paul, Minn.

WEDNESDAY AFTERNOON, SEPTEMBER 12th
2:00 P. M. to 5:00 P. M.

"The Utility of Radiography in Operative Dentistry with Special Application to Preventive Service," by Clarence O. Simpson, St. Louis, Mo.

Synopsis—Adequate standards, and how they are attained. The advantages of vigilance and early diagnosis. The value of routine radiodontic examinations. Indications in secondary dentition. The detection of incipient caries and periodontal lesions. Radiographic assistance in restorative procedures. Radiographic service in pulpless teeth. Refinement and judgment in the interpretation of radiographic evidence.

Discussed by C. Edmund Kells, New Orleans, La.; Howard R. Raper, Albuquerque, New Mex.

"The Value of Antiseptics in the Root-canal Filling," by Arthur B. Crane, Washington, D. C.

Synopsis—The natural defensive processes indicate the rationality of root-canal work. Present diagnostic methods are unreliable in periapical disease. The restoration of the periapical norm is attainable in certain cases of periapical disease; and by every standard of pathology this is evidence of cure. The total obliteration of the pulp channels is impossible by any present technic but such obliteration is not necessary.

Discussed by Carl J. Grove, St. Paul, Minn.; H. D. Grubb, Cleveland, Ohio.

THURSDAY MORNING, SEPTEMBER 13th
9:00 A. M. to 12:00 M.

"A Consideration of the Effects of Trauma Upon the Periodontal Tissues Produced by Indifferently Executed

Restorations,'' by Frederick A. Bricker, Rochester, Minn.

Synopsis—How trauma is caused through the use of hard gold having greater resistance to wear than the tooth structure. Objections to the use of wax and carbon paper in correcting occlusion. Direction of stress during mastication and while jaws are at rest or in centric occlusion. Method of diagnosing and correcting traumatic occlusion.

Discussed by Frank H. Skinner, Chicago, Ill.; George R. Ellis, Washington, D. C.

''Periodontal Diseases and Their Treatment from the Viewpoint of Operative Dentistry,'' by F. W. Merrifield, Chicago, Ill.

Synopsis—Review of the structures composing the investing tissues of the teeth, their histology, physical functions and the possibilities of their recuperation following disease or accident. The importance of correct operative procedures, not only as to prevention, but also palliative measures and cure will be emphasized. A conservative outline for the general practitioner rather than the specialist.

Discussed by John F. Biddle, Pittsburgh, Pa.; J. H. Phillips, Nashville, Tenn.

### Full Denture Prosthesis

(Rainbow Room, Winton Hotel. All subsequent meetings will be held in the same place.)

Officers of Section—H. B. Washburn, Chairman, St. Paul, Minn.; I. L. Furnas, Vice-Chairman, Cleveland, Ohio; Victor H. Sears, Secretary, Salt Lake City, Utah.

### Tuesday Afternoon, September 11th
### 2:00 P. M. to 5:00 P. M.

(This section program is arranged with the thought that consideration of a subject should begin with fundemantals and proceed from that point in logical sequence. Subjects were selected and then the essayists invited who were qualified to present the subject.)

"Regional Anatomy, Emphasizing Mandibular Movements with Specific Reference to Full Denture Construction," by H. J. Prentiss, Iowa City, Ia.

Synopsis—(1) A general consideration of the mandible and maxilla from birth to adult life in human type. (2) Comparison with lower forms. (3) Presence of external pterygoid plate in human type. Its absence in lower forms. (4) Accentuation of eminentia articularis in human type. (5) Digastricus in lower types. Modifications in human type. (6) Spheno-meniscus muscle in humans and lower types. (7) External pterygoid in human type. Absence in lower types. (8) Reasons for presence of meniscus in all types. (9) Its peculiar significance in human types. (10) Action of internal pterygoids. Action of spheno-meniscus. Action of external pterygoid in human. (11) Destruction of meniscus in human following loss of teeth. (12) Possible compromise in adjustments of plates. Possible complications. (13) Mechanism of mandibular ligaments in human type. (14) Summary.

Discussed by W. H. Wright, Pittsburgh, Pa.

"Mechanics of Denture Construction," by I. M. Hair, Atlanta, Ga.

Synopsis—The mechanics of full denture construction consists of five points of consideration, and includes the factors of retention, balance, condyle relation to glenoid fossae, and vulcanization.

1. Contour and form of the arch in relation to retention factors: (a) Adhesion. (b) Cohesion. (c) Atmospheric pressure. (d) Outline of denture to points of muscle attachments and soft tissue. Ople's Law of Gases. (e) Reliefs for median line, etc.

2. Registration of Central Occlusion: (a) Condyles in glenoid fossae. (b) Pressure equalization. (c) Freedom of tongue and lips.

3. Position of Teeth on Ridge: A. Leverage. (1) Fulcrum Point. (a) Normal thickness of mucosa. 1. Creat of ridge if relieved, otherwise median raphe of palate.

(b) Soft, flabby ridge. (a) Junction of hard and soft area. (b) Materials. (1) Artificial stone. (a) Proper consistency. (b) Method of packing the stone. (c) Requirements of a stone. Non-expanding, non-disintegrating in vulcanizer. (2) Gold or Vulcanite. (c) Removing wax. (d) Waste grooves. (e) Packing. (f) Testing (1) Proper excess. (g) Pressed. (h) Vulcanization (1) Temperature. (2) Steam vulcanizing. (i) Opening and cleaning cases and trimming cases. (j) Finishing and polishing. (k) Remounting. (b) Tongue Space. (1) Should not crowd tongue lingually. C. Relation to cheek tissues. (1) Teeth should not be too far buccally because of leverage; because of destroying face form and causing cheek biting.

4. Cusp Co-ordination or articulation. (1) Central Occlusion. (2) Working Occlusion. (3) Balancing Occlusion. (4) Incising Occlusion. (a) Prevents rotation and tripping which is followed by irritation and absorption.

5. Investing, packing, vulcanizing and finishing case. (a) Flask type. (1) Milled edges for accuracy in closing. (2) Pressure Type. (a) Allows expansion of vulcanite without distortion.

Discussed by W. F. Lasby, Minneapolis, Minn.; Edwin H. Mauk, San Francisco, Cal.

WEDNESDAY MORNING, SEPTEMBER 12th
9:00 A. M. to 12:00 M.

"The Fundamental and Basic Principles of Mouth Classification," by M. M. House, Kansas City, Mo.

This will cover the same, beginning with biological development and showing the true conditions as applied to edentulous conditions due to modifications caused by surgical interference and absorptions. It will also show some of the principles of applied physics and art.

Discussed by Geo. P. Brenner, Milwaukee, Wis.; R. O. Schlosser, Chicago, Ill.

"History and Evolution of Occlusion," by H. B. Washburn, St. Paul, Minn.

Synopsis—For the purpose of better presentation we have specified three periods. First, primitive dentistry. (This includes everything before Bonwill's time.) Second, Bonwill Era (1850-1890). Third, Renaissance period of dentistry. (Beginning in 1890 with Walker presenting a new idea which has influenced all theories on occlusion since.) We shall attempt, by setting down in sequence the high lights in the history of this subject, to demonstrate that present day theorems on occlusion are a natural evolution.

Discussed by Wm. A. Giffen, Detroit, Mich.; O. DeForest Davis, Minneapolis, Minn.

### Wednesday Afternoon, September 12th
### 2:00 P. M. to 5:00 P. M.

"Esthetics," by J. W. Crawford, Milwaukee, Wis.

Synopsis—The anatomical relation of the muscles to mastication and expression, the relationship of the mandible to the maxilla and the position of the condyles in the glenoid fossa as a basis for esthetics in full dentures.

Discussed by R. G. Keyworth, St. Paul, Minn.; P. C. Lowery, Detroit, Mich.

"The Masticatory Mechanism Considered in its Relation to the Chemistry and Mechanics of Digestion," by Thomas B. Hartzell, Minneapolis, Minn.

Synopsis—I will treat briefly the development and use of the natural teeth as they affect the bones of the jaw itself, and the bony structure of the base of the skull; the effect of mastication in developing the strong lines in the support of the teeth; the effect of mastication in developing the salivary glands; the relation of salivary digestion to the stomach and intestinal digestion; the value of correct and efficient mastication in its relation to the digestion of foods and finally a consideration of the artificial denture in restoring natural and efficient mastication and a consideration of advantages that arise from the replacement of efficient masticating apparatus by imperfect artificial dentures as compared to the more perfect modern type of denture now being evolved by the prosthetist.

Discussed by Wallace Seccombe, Toronto, Canada; Thomas P. Hinman, Atlanta, Ga.

"Bacterial Growths on Artificial Dentures," by W. J. Pryor, Cleveland, Ohio.

Synopsis—1. A review of work done by men before me on the inhibitory action upon bacterial growths of various materials that may be used in the construction of artificial dentures. 2. A report of the work done in this field by the essayist. 3. Suggestions that may be helpful to those who would construct dentures that will tolerate the mouth tissues with the least amount of irritation.

Discussed by Ira G. Nichols, Chicago, Ill.

THURSDAY MORNING, SEPTEMBER 13th
9:00 A. M. to 12:00 M.

"Case Histories," by Geo. S. Monson, St. Paul, Minn.

Synopsis—1. The history of four cases that occur in general practice which would be the same as many others that confront us. 2. The placing of the mandible in a correct relation to the other bones by its muscular balance. 3. The unrolling of facts that were not anticipated in each case. 4. The results in general health in each of the cases.

Discussed by Frank H. Skinner, Chicago, Ill.; William Denton Taylor, Newark, N. J.

"Cusp Trauma," by Felix A. French, Ottawa, Canada.

Synopsis—1. Its vital importance in full denture construction. 2. Cusp trauma as a cause of tissue atrophy. 3. Difference between cusp trauma in natural and artificial dentition. 4. Factors governing cusp form and arrangement. 5. Relation between cusp form and ridge form.

Discussed by Rupert E. Hall, Chicago, Ill.; F. M. Hight, Houston, Texas.

"The Dentist's Duty to His Edentulous Patients," by Robert H. Gillis, Hammond, Ind.

Synopsis—His preliminary training, mental and technical; The Patient's dire need; Case analysis and diagnosis; Prognosis and education of the patient; Demonstration of technical ability; Co-operation essential; Analysis of result; Summary.

Discussed by Victor H. Sears, Salt Lake City, Utah; J. B. La Due, Chicago, Ill.

### Partial Denture Prosthesis

(Main Ballroom, Winton Hotel. All subsequent meetings held in same place.)

Officers of Section—Theodore W. Maves, Chairman, Minneapolis, Minn.; Willis A. Coston, Vice-Chairman, Topeka, Kansas; O. DeForest Davis, Secretary, Minneapolis, Minn.

### Tuesday Afternoon, September 11th
### 2:00 P. M. to 5:00 P. M.

"Balanced Occlusion and Its Relation to Partial Denture Construction," by J. W. Needles, Pueblo, Colorado.

Synopsis—1. Definition. 2. Distinction from traumatic occlusion. 3. Fundamental to all branches of dentistry. 4. Ideals of normal occlusion. 5. Mechanism of maxillo-mandibular joint. 6. Distribution of the forces of mastication. 7. Relation of cusp depth and overbite to curve of Spee. 8. The Spherical theory. (a) Opening closing axis. (b) Varying axes of lateral and protrusive movement. (c) "Rotation Center." (d) Components of movement at condyle and incisal point. (e) Development of maxillary apparatus. (f) Spherical theory predicts results of new conditions. (g) Spherical theory as a foundation for accurate technic. (h) Central occlusion. (i) Range of occlusion. (j) Restoration of ideal conditions. (k) Choosing new central occlusion. 1. Degree of accuracy necessary. 9. Therapeutics. (a) Do we wish to conform to existing conditions? (b) Do we wish to modify for mechanical or therapeutic reasons? (c) Do we wish to modify to establish ideal relations?

WEDNESDAY MORNING, SEPTEMBER 12th
9:00 A. M. to 12:00 M.

"Conditions Affecting the Solution of the Esthetic Problem in Fixed Bridgework," by Forrest H. Orton, St. Paul, Minn.

Synopsis—1. Differentiation in the esthetic requirement of the various types of partial denture restoration. 2. Differentiation in the esthetic requirements of the upper and lower teeth. 3. Characteristics of remaining natural teeth: (a) Anatomy. (b) Pathological scars, Abrasion, Erosion. (c) Age, Checks, Stain. (d) Alignment, etc. 4. Methods of studying line employed in art schools: (a) Color considerations natural teeth: (What each tissue contributes: Enamel, dentin.) Effect of environment: Lips, oral cavity, reflection. 5. Comments on flat back teeth furnished by the manufacturer with recommendations. 6. Conclusion, with suggestions in teaching technic of matching natural teeth.

"The Reasons for, and a Technic for, Obtaining Balanced Occlusion in Fixed Reconstruction." by C. K. Bird, St. Paul, Minn.

Synopsis—This paper will feature some of the fundamental factors in fixed reconstruction. What balanced occlusion is, why necessary, and with the aid of slides attempt. to show a method for restoring balanced occlusion in mutilated arches.

WEDNESDAY AFTERNOON, SEPTEMBER 12th
2:00 P. M. to 5:00. P. M.

"Preparations Indicated on the Various Types of Teeth for Bridge Abutments, Preserving Vitality," by Theo. W. Maves, Minneapolis, Minn.

"Removable Bridgework," by B. B. McCollum, Los Angeles, Cal.

THURSDAY MORNING, SEPTEMBER 13th
9:00 A. M. to 12:00 M.

"The Cast Gold Abutment in Combination With a Porcelain Jacket Crown in Bridge Restorations," by J. E. Argue, Seattle, Wash.

Synopsis—It will consist of an illustrated paper, describing and illustrating the different steps in the technic of the operation, also illustrations showing completed cases demonstrating the highly artistic result that can be obtained in the mouth.

"Fixed Bridgework," by Emory C. Thompson, Warren, Pa.

Synopsis—Problems and important factors involved in manipulation of wax, investing process, carbonization and elimination of wax in the burning-out process, casting and a few fundamentals pertaining to metallurgy.

ORAL SURGERY, EXODONTIA AND ANESTHESIA
(Ballroom, Hollenden Hotel. All subsequent meetings held in same place.)

Officers of Section—J. P. Henahan, Chairman, Cleveland, Ohio; Boyd S. Gardner, Vice-Chairman, Rochester, Minn.; Frederick F. Molt, Secretary, Chicago, Ill.

TUESDAY AFTERNOON, SEPTEMBER 11th
2:00 P. M. to 5:00 P. M.

"Shock," by George W. Crile, M. D., Cleveland, Ohio.

Discussed by Truman W. Brophy, Chicago, Ill.; Don M. Graham, Detroit, Mich.

"Alveolar Process Resection," by Carl D. Lucas, Indianapolis, Ind.

Synopsis—Purpose, prosthetic restoration requirements. Elimination of oral foci of periapical infection. Care of isolated areas of pathologic lesions. Importance of unbroken chain of asepsis. Considerations in bone surgery. Anesthetics. Care of patients pre-operatively and post-operatively if general anesthesic is to be employed. Hospitalization of these operative cases always appreciated by the patients. Preparation of the field of operation. Manipulative technic of complete and partial upper resections. Manipulative technic of complete and partial lower resections. Resections frequently necessary as a post-extraction requisite for cosmetic denture construction. Resections contraindicated in many cases of extraction. Study models necessary. Suturing. Ridge requirements. Muscular attachments. Healing and resultant shrinkage. Scar tissue density. Temporary denture restorations. Secondary denture restorations. Osseous resorption following resection. Co-operation with the prosthetist in all cases.

Discussed by Frederick B. Moorehead, Chicago, Ill.; Frederick F. Molt, Chicago, Ill.

WEDNESDAY MORNING, SEPTEMBER 12th
9:00 A. M. to 12:00 M.

"Plastic, Facial and Maxillary Surgery," by G. V. I. Brown, Milwaukee, Wis.

Synopsis—Widened experience has taught plastic surgeons that many more or less spectacular operative undertakings are really very simple and with ordinary skill and care almost uniformly successful. Stereopticon illustrations covering a wide variety of facial reconstructive operations will be shown to present as clearly as possible the reasons for the plastic operative precepts that are recommended and to show the results of their application.

Discussed by Chalmers J. Lyons, Ann Arbor, Mich.; William L. Shearer, Omaha, Nebraska.

"Importance of Technic in Administering Nitrous Oxid and Oxygen Anesthesia," by C. K. Teter, Cleveland, Ohio.

Synopsis—Of all the general anesthetics, Nitrous Oxid is the most difficult to administer and requires a nearer perfect technic than any of the others. The vast majority of all complications encountered when administering Nitrous Oxid and Oxygen are due to faulty technic.

It has often been said by the operating surgeon that a good anesthetist is born, not made. It is just as essential in this specialty as in any other specialty of medicine or dentistry that one who enters it should be endowed with special qualifications and aptitude along this line. A successful anesthetist is an artist as well as a scientist. He must be endowed with quick perception, quick diagnostic ability and a thorough knowledge of physiology, anatomy and pathology. He must be familiar with the scientific and chemical reactions produced by the agents used. There is no short-cut to real success and expertness in any line without a great deal of study and hard work. The day of the overnight specialist is, or should be past, for such are not deserving of support from the professions. There is a crying need for competent post-graduate instruction in this line of work, open to all who may desire it.

Discussed by B. H. Harms, Omaha, Nebraska; J. A. Heidbrink, Minneapolis, Minn.; E. I. McKesson, M. D., Toledo, Ohio.

WEDNESDAY AFTERNOON, SEPTEMBER 12th
2:00 P. M. to 5:00 P. M.

"Focal Infection and Dental Diseases." by Edward H. Hatton, M. D., Chicago, Ill.

Synopsis—In chronic alveolar abscess sections through the diseases apices and the investing bone. Show changes which will be demonstrated by micro-photographs and specimen. The relationship of these changes to dissemination of infection will be discussed. In addition the factors of importance in arriving at a diagnosis of focal infection will be appraised, especially such laboratory tests as blood analysis.

Discussed by John G. Meisser, Rochester, Minn.; T. A. Hardgrove, Fond du Lac, Wis.

"Foreign Bodies of Dental Origin in Air and Food Passages," by Carlos E. Pitkin, Cleveland, Ohio.

Synopsis—Foreign bodies encountered in the air and food passages, from the oral cavity and as a result of oral surgical procedures. Prophylaxis, localization and methods of removal. Illustrative cases. Lantern slides.

Discussed by Claire L. Straith, Detroit, Mich.; Joseph P. Wahl, New Orleans, La.

"Atypical Neuralgias and Their Treatment," by S. L. Silverman, Atlanta, Ga.

Synopsis—A brief presentation of method of treatment through the use of cantharides, by which gratifying results have been obtained in cases of head pains not of the tic type.

THURSDAY MORNING, SEPTEMBER 13th
9:00 A. M. to 12:00 M.

"Surgery of the Antrum of Highmore," by Virgil Loeb, St. Louis, Mo.

Synopsis—Anatomical relationship of the Antrum of Highmore to the mouth and nose. Consideration of variations in size and shape in the same individual. Etiology and diagnosis of antral diseases which may require surgical interference. Indications and contraindications for such interference. Types of operations.

Discussed by Theodor Blum, New York, N. Y.; M. N. Federspiel, Milwaukee, Wis.

"Cysts and Tumors of the Jaws," by Henry S. Dunning, New York, N. Y.

Synopsis—1. Their occurrence. Frequency, site, chararteristics. 2. Etiology. Predisposing causes of age, sex, etc. The dental surgeon's responsibility. 3. Pathology of; Variations of; Classification. 4. Diagnosis. Methods of establishing same. Interpretation of X-rays. 5. Treatment of; When to operate. (a) Operative Method of operation. (b) Non-operative. Anesthesia. 6. Prognosis.

Discussed by Rea Proctor McGee, Pittsburgh, Pa.; Arthur E. Smith, Chicago, Ill.

ORTHODONTIA AND PERIODONTIA

(Ballroom, Statler Hotel. All subsequent meetings held in same place.)

Officers of Section—Clyde M. Gearhart, Chairman, Washington, D. C.; William C. Fisher, Vice-Chairman, New York, N. Y.; B. Frank Gray, Secretary, San Francisco, Cal.

TUESDAY AFTERNOON, SEPTEMBER 11th
2:00 P. M. to 5:00 P. M.

"The Alimentary Tract, a Talk to Dental Patients," by Gillette Hayden, Columbus, Ohio.

Synopsis—The mouth the first of three reservoirs. Its strategic position as the port of entry; its service to the whole body; its share in the choice of foods and in their preparation for the second revervoir, the stomach. The journey of the body's fuel from the second to the third reservoir or colon. Some of the effects of an imperfectly used or a crippled dental apparatus upon the other parts of the alimentary tract.

Discussed by Celia Rich, Nashville, Tenn.; E. Melville Quinby, Boston, Mass.

"Clinical Evidence, with a Working Hypothesis, Favoring Pulp Extirpation in Children's Teeth," by Norris C. Leonard, Baltimore, Md.

Synopsis—For orthodontic reasons, alone, continued healthy functioning of the deciduous teeth, and the preservation of the permanent ones erupted during the developmental period of the jaws is of immense importance. A simple method is presented for consideration of both the general practitioner and the orthodontist for treatment of pulp exposures, favoring the physiological shedding of the deciduous teeth, and continued development of the incompletely calcified roots of the permanent ones.

Discussed U. G. Rickert, Ann Arbor, Mich.; Harry E. Kelsey, Baltimore, Md.

"The Varied Reactions to Injury from Traumatic Occlusion," by Paul R. Stillman, New York, N. Y.

Synopsis—The paper discusses and differentiates between stimulation and irritation. The phenomenon of irritation being the fundamental factor in the etiology of inflammation, viz., "a reaction to injury." Disease of the peridontium and allied structures is described as an inflammation. Several very unusual cases are described and discussed, the cases being selected with the intention of showing "the varied reactions to injury from traumatic occlusion." This relation of the teeth is identified as a mechanical injurious agent whose influence is at times a stimulant and induces inflammation according to the degree of resistance to attack. The subject is also discussed as to whether traumatic occlusion occurs as a result of migration due to inflammation or if it is a constant factor in those cases having it, and is thereby of itself the cause of disease.

The relation of traumatic occlusion as it has been observed when induced by the migration of teeth through orthodontic treatment is discussed. A distressing case illustrating such an instance is described. Slides of clinical cases will be shown.

Discussed by Frank M. Casto, Cleveland, Ohio; Olin B. Kirkland, Montgomery, Ala.

WEDNESDAY MORNING, SEPTEMBER 12th
9:00 A. M. to 12:00 M.

"The Nomenclature of Periodontology," by John Oppie McCall, Buffalo, N. Y.

Synopsis—Periodontology is a branch of dentistry whose development has been very rapid in the past ten years. With increasing knowledge there has come the need for a terminology adequate to express this knowledge. The terms presented in this paper are largely those which have been adopted by the American Academy of Periodontology. Many of these are in use in the dental literature of today. But it has been thought advisable to present a somewhat extended discussion of these words, to make clear their correct usage, and to give opportunity for debate as to their suitability.

Discussed by R. Ottolengui, New York, N. Y.; L. Pierce Anthony, Philadelphia, Pa.

"Bridgework from a Periodontist's Standpoint," by Andrew J. McDonagh, Toronto, Canada.

Synopsis—The effect of artificial substitutes for teeth which have been lost as far as the investing tissues of the other teeth are concerned. The reasons given for the injurious torque. How torque acts on the investing tissues of the natural teeth; why pocket formation takes place; why so-called recession of the gingival tissues takes place; why one kind of artificial restoration has less injurious effect on the periodontal tissues than another kind. How fixed bridgework compares with removable bridgework in this connection and the best method, from the standpoint of preservation of the periodontal tissues. The best method of replacement from the standpoint of periodontal tissues.

Discussed by Arthur H. Merritt, New York, N. Y.; Justin D. Towner, Memphis, Tenn.

To the memory of Calvin S. Case, who, life permitting, would have occupied this hour with a valuable presentation on "The Early Regulation of Children's Teeth."

"Calvin S. Case, The Teacher"—John P. Buckley; "Calvin S. Case, The Author"—Otto U. King; "Calvin S. Case, The Practitioner and Scientist"—Hart J. Goslee; "Calvin S. Case, The Man"—C. N. Johnson; "Calvin S. Case, The Orthodontist and Friend"—Thos. L. Grisamore.

WEDNESDAY AFTERNOON, SEPTEMBER 12th
2:00 P. M. to 5:00 P. M.

Wednesday afternoon to be devoted to program of Detroit Clinic Club.)

THURSDAY MORNING, SEPTEMBER 13th
9:00 A. M. to 12:00 M.

"Orthodontic Eruption of Impacted Cuspids and Incisors," * by Varney E. Barnes, Cleveland, Ohio.

Synopsis—1. Expand to provide *extra space* for impacted tooth. 2. Fit removable retainer as anchorage.

Add springs and hooks to hold surgical packing. 3. Surgical exposure of impacted tooth. 4. Fit packing of paraffin saturated spunk over tooth to fill wound. Attach to retainer. Cover packing with antiseptic powder and lock appliance in place. 5. Apply antiseptic and replace frequently until wound heals, exposing tooth. 6. Band tooth if possible, or drill pit for special locked inlay, carrying loop or hook. 7. Erupt tooth with orthodontic pressure.

Discussed by Herbert A. Pullen, Buffalo, N. Y.; Burt Abell, Toledo, Ohio.

"Endocrine Activity as Applied to Orthodontic Types of Unknown Origin," by C. C. Howard, Atlanta, Ga.

Synopsis—A general consideration of bony development viewed from the standpoint of ductless gland disorders. Association of dental arch and jaw mal-formation is studied in the correlation of such disturbances. An attempt is made to definitely connect these phenomena, considering the possibility of linking together an imbalance of the glandular system with improper arch progress.

Discussed by Albert W. Crosby, New Haven, Conn.; Joseph D. Eby, New York, N. Y.

### Mouth Hygiene and Preventive Dentistry

(Arena, Public Hall. All subsequent meetings held in same place, except Thursday A. M., which will be held in Lounge, Public Hall.)

Officers of Section—Wm. H. Card, Chairman, Minneapolis, Minn.; L. G. Mitchell, Vice-Chairman, Los Angeles, Cal.; Thos. B. McCrum, Secretary, Kansas City, Mo.

### Tuesday Afternoon, September 11th
### 2:00 P. M. to 5:00 P. M.

"The Problem of Introducing Oral Hygiene Into the Public Schools as a Course of Study," by R. S. Towne, Bismarck, N. D.

Synopsis—1. Co-operation of the educational authorities with the dental association. 2. Instruction of teachers and school nurses. 3. Supplying texts for use in teaching the subject.

Discussed by Earl S. Braithwaite, Willard, Ohio.

"Marion School Class After a Dozen Years," by Cordelia O'Neill, Cleveland, Ohio.

(Some of the Marion School Class, two of whom are now dentists will be present at this meeting.)

Discussed by Leonard G. Mitchell, Los Angeles, Cal.

"Publicity Methods in Oral Hygiene," by Rea Proctor McGee, Pittsburgh, Pa.

Discussed by Sidney J. Rauh, Cincinnati, Ohio.

"Scope and Phases of Preventive Dentistry from the Forsyth Clinics," by Harold DeWitt Cross, Boston, Mass.

Discussed by Thos. B. McCrum, Kansas City, Mo.

"The Establishment of a Mouth Hygiene Division in State Departments of Health," by Taliaferro Clark, M. D. (U.S.P.H.S.), Washington, D. C.

Discussed by Alfred C. Fones, Bridgeport, Conn.

WEDNESDAY MORNING, SEPTEMBER 12th
9:00 A. M. to 12:00 M.

"Results Obtained by Five Years' Thorough Dental Work for School Children of West Virginia" (With Motion Pictures), by G. T. Epling, Welch, W. Va.

Discussed by E. L. Pettibone, Cleveland, Ohio; Geo. C. Howard, West Union, W. Va.

"Nutrition and Function in Relation to the Teeth," by Wallace Secoombe, Toronto, Canada.

Discussed by Clarence J. Grieves, Baltimore, Md.; Harold DeWitt Cross, Boston, Mass.

"Who is an Oral Hygienist," by Evelyn C. Schmidt, Boston, Mass.

Discussed by Harold J. Leonard, Minneapolis, Minn.; Gladys Eyrich, Jackson, Miss.

"Proposed State Training School for Hygienists," by A. C. Fones, Bridgeport, Conn.

Discussed by Guy S. Millberry, San Francisco, Cal.

"Then Dental Hygienist, Her Place in Schools; Industry; Hospital Service; and in the Private Dental Office,'' by C. J. Hollister, Harrisburg, Pa.

Discussed by Henry L. Banzhaf, Milwaukee, Wis.

WEDNESDAY AFTERNOON, SEPTEMBER 12th
2:00 P. M. to 5:00 P. M.

''Sope of Work in an Industrial Dental Clinic,'' by R. W. Elliott, M. D., Cleveland, Ohio.

Discussed by H. M. Brewer, Dayton, Ohio.

''Dental Education and Industrial Dental Clinics,'' by H. L. Banzhaf, Milwaukee, Wis.

Discussed by A. G. Buckner, Nashville, Tenn.

''Methods of Teaching Oral Hygiene with Lesson Outlines,'' by John P. Erwin, Perkaskie, Pa.

Discussed by Rea Proctor McGee, Pittsburgh, Pa.

THURSDAY MORNING, SEPTEMBER 13th
9:00 A. M. to 12:00 M.

''Diet in its Relation to Growth and Development, with Special Relation to the Teeth,'' by John E. Gurley, San Francisco, Cal.

Synopsis—(a) The pregnant mother. (b) From birth to two years.

Discussed by Wallace Seccombe, Toronto, Canada.

''Periodontia—What is it?'' by Philip R. Thomas, Minneapolis, Minn.

Discussed by John E. Gurley, San Francisco, Cal.

''Dentistry for Children, Development and Care of Same,'' by Richard A. Suhr, Cleveland, Ohio.

Discussed by M. Evangeline Jordon, Los Angeles, Cal.

''The Care with Filling Procedure of Deciduous Teeth and the First Permanent Molar,'' by Wm. Z. Hill, Philadelphia, Pa.

Synopsis—Starting time, examinations, talk to patient, cavity preparation, filling material, treatment of involved pulps.

Discussed by Walter Liles, Richmond, Va.

''Oral Hygiene in the Bridgeport Schools,'' by Mrs. Hubert W. Hart, Bridgeport, Conn.

Discussed by Philip R. Thomas, Minneapolis, Minn.

HISTOLOGY, PHYSIOLOGY, PATHOLOGY, BACTERIOLOGY AND CHEMISTRY (RESEARCH)

(State, Public Hall. All subsequent meetings held in same place.)

Officers of Section—Weston A. Price, Chairman, Cleveland, Ohio; Julio Endelman, Vice-Chairman, Los Angeles, Cal.; Harris R. C. Wilson, Secretary, Cleveland, Ohio.

TUESDAY AFTERNOON, SEPTEMBER 11th
2:00 P. M. to 5:00 P. M.

A Symposium on "Evidences For and Against Nutrition, or Calcification Changes of Enamel After Tooth Eruption."

1. Newer Histological Data on the Structure of Enamel, by J. Leon Williams, New York, N. Y.

2. Enamel Rod Sheaths, the End Organs of the Dentoenamel Circulation, by Charles F. Bodecker, New York, N. Y.

3. Changes of Enamel After Tooth Eruption, by U. G. Rickert, Ann Arbor, Mich.

4. Physiological Changes in the Structure of the Enamel After Tooth Eruption, by Theodor B. Beust, Louisville, Ky.

5. Permeability of Enamel, by William G. Skillen, Chicago, Ill.

---

WEDNESDAY MORNING, SEPTEMBER 12th
9:00 A. M. to 12:00 M.

A Symposium on "Factors Constituting Potential Dangers from Dental Infection."

1. Evidences of the Relation Between Systemic Involvements and Dental Foci, by John G. Meisser, Rochester, Minn.

2. Calcification Within the Canals of Teeth, by William G. Skillen, Chicago, Ill.

3. Histopathology of Teeth and Associated Structures in Situ with Case History, Radiographic, and Post-

Mortem Findings, by Karl Meyer, M. D., Chicago, Ill., and C. S. Suddarth, Chicago, Ill.

4. Dentistry's Relation to Psychopathology, by E. A. Thomas, Hastings, Neb.

WEDNESDAY AFTERNOON, SEPTEMBER 12th
2:00 P. M. to 5:00 P. M.

Miscellaneous Research Reports.

1. The Importance of the Wetting Phenomena in Bacteriology, by W. P. Larson, M. D., Minneapolis, Minn., and T. B. Hartzell, Minneapolis, Minn.

2. Report on Decalcification of Enamel, by Thomas B. Hartzell, Minneapolis, Minn.

3. Renal Changes in Primary Hypertention, by E. T. Bell, M. D., Minneapolis, Minn.

4. The Effects of Nutritional Disturbances Upon Calcification and Decalcification of Teeth in Experimental Animals, by John A. Marshall, San Francisco, Cal.

5. Further Studies in the Physiological and Pathological Variations in the Composition of Human Saliva, by Henry C. Ferris, New York, N. Y.

THURSDAY MORNING, SEPTEMBER 13th
9:00 A. M. to 12:00 M.

A Symposium on "The Nature of Defensive Forces Preventing Each (a) Dental Caries, (b) Periodontoclasia, (c) Systemic Involvements from Dental Infections."

1. Some Experimental Effects of Deficient Diets on the Monkey, by Percy R. Howe, Boston, Mass.

2. Comparative Analyses of Blood and Saliva, with Special Reference to Such Substances as Sugar, Urea, Calcium and Other Mineral Constituents, by Edw. H. Hatton, M. D., Chicago, Ill.

3. The Relationship Between Clinical Evidence and Blood Chemistry in Focal Infections, by B. J. Bixby, M. D., Buffalo, N. Y.

4. Calcium Metabolism in Relation to Susceptibility to and Defense Against (a) Dental Caries, (b) Periodontoclasia, (c) Systemic Involvements from Dental Infections, by Weston A. Price, Cleveland, Ohio.

### The Clinic Program

The clinic program for the Cleveland meeting, in so far as it has been completed to date, is herewith submitted. All clinics will be given on Thursday afternoon or Friday morning. You will note that in order to insure an equitable distribution of clinicians, the allotment of clinics awarded to the several states has been in accordance with the membership in the American Dental Association, and is based upon state representation in the House of Delegates.

No clinician has been accepted without the indorsement of a committee from his state society, assuring the membership of the selection of outstanding talent to represent each component organization.

It will be noted that a number of states have neglected to respond to the invitation up to this time, but we believe that much of the work now under way will result in the completion of their quota within the next few weeks. It is hoped that the names of desirable clinicians holding membership in states falling below their quota in this issue will be forwarded to the presidents of their respective state societies in order that their services may be secured in time for publication in the program. Section clinics will be given in conjunction with the general clinics by essayists who desire to supplement the papers which they have presented before their respective sections. Thus will the subject-matter of section discussions become available to the entire membership.

Groups and study clubs will occupy another section and will not be counted under the state limitation; but no state will be permitted to furnish more than three groups in addition to their authorized quota of individual clinicians.

The Ohio State Dental Society, acting as our host, will furnish a general program of fifty clinicians and will occupy a section.

All who have seen the magnificent auditorium in Cleveland will readily appreciate our opportunity for staging a wonderful clinic. Special booths now under construction will bear placards of the states represented and will afford amply opportunity for each individual clinic to be seen and fully understood without interruption. Don't miss the clinics.—P. G. Puterbaugh, General Chairman of Clinics.

General and Group Clinics Classified by States

*Alabama*—(Expected to furnish three clinicians.)

*Alaska*—(Expected to furnish two clinicians.)

*Arkansas*—(Expected to furnish three clinicians.)

"Care of the Gingival Crevice in Prophylaxis and Pyorrhea." Mounted specimen together with illustrations demonstrating normal and pathological conditions of teeth, and gum tissue. Proper method of brushng and instrumentation. By J. E. Crozier, Little Rock.

"The Practical Application of the Cast Clasp in Partial Denture and Bridge Construction." A technic for the accurate construction of cast clasps and a demonstration, with models, of the application of the cast clasp to partial denture and bridge construction." By M. H. Gray, Little Rock.

"A Standardized Technic for Casting Using the Same Principles From the Inlay to the Gold Plate."

(*Group Clinic*)

A. Boil-out technic for elimination of wax.

B. Casting dentures and gold bases for full cases, by F. G. White, Fort Smith.

C. Casting inlays and three-quarter crowns in thirty minutes, by T. J. Newman, Little Rock; J. D. Jordon, Little Rock; E. L. Miley, Fort Smith.

*Arizona*—(Expected to furnish two clinicians.)

*California State*—(Expected to furnish eight clinicians.)

"Porcelain Jacket Crowns," by Hugh Avary, San Francisco.

"Continuous Gum Dentures," by James Allen Graham, San Francisco.

"Pyorrhea Surgery and Protection of Mouth Wounds." Using a skull instead of patient the various steps of alveolar curettage, gum excision and the protection of the wound will be demonstrated. By A. W. Ward, San Francisco.

*California*—"Orthodontia," by B. Frank Gray, Berkeley.

*Colorado*—(Expected to furnish five clinicians.)

"Alveolotomy," by M. R. Howard, Denver.

"Incisions and Flap Formations for Oral Surgery." Demonstrated on Models and Skull. By John W. Seybold, Denver.

*"Prosthesis"*

1. Preparation to show that spherical surfaces allow balanced relation in all positions.

2. Spherical bite method showing central occlusion and range of occlusion.

3. Preparation showing relation of curve of Spee to condyle path and the depth of cusp and overbite.

4. Preparation showing that central occlusion may be established at any point within the range of occlusion by proper arrangement of inclined planes, by J. W. Needles, Pueblo.

*Connecticut*—(Expected to furnish five clinicians.)

"Some Bridge-Attachments," by J. D. Hertz, Stanford.

"Heat-Treatment of Platinum Gold Alloys," by F. T. Murlless, Jr., Hartford.

"Definite Technic for Localizing Impacted Lower Third Molars," by George C. Fahy, New Haven.

"Partial Compound Impressions," by E. Frank Cory, New Haven.

"Spring Attachments for Clasps on Lingual Bar Plates," by Morton J. Loeb, New Haven.

*Delaware*—(Expected to furnish two clinicians.)

*District of Columbia*—(Expected to furnish three clinicians.)

"Preparation For and Construction of Three-Quarter Cast Crown," by Vernon J. Lohr, Washington.

*Florida*—(Expected to furnish three clinicians.)

*Georgia*—(Expected to furnish five clinicians.)

*Hawaiian Islands*—(Expected to furnish two clinicians.)

*Idaho*—(Expected to furnish two clinicians.)

*Illinois*—(Expected to furnish twenty-three clinicians.)

"Reproducing Plates Made Too Soon After Extraction," by H. O. Browning, Chicago.

"Prevention in Dentistry." Prevention of Caries by (1) prophylactic procedures; (2) by chemical application; (3) by cements. Prevention of Malocclusions by (1) early diagnosis; (2) by early radiographic interpretation; (3) by space retainers. By F. Blaine Rhobotham, Chicago.

"Cast Gold Inlays." Finished and unfinished cast gold inlays using a wax eliminator. By A. P. Grunn, Chicago.

"Closing Persistent Fistulas Between Antrum and Mouth." Showing various means of closing these openings from simple freshening of surfaces to the moving of tissue flaps and the lines of incision for these flaps. By Earle H. Thomas, Chicago.

"Casting of Porcelain." Casting of Porcelain using an investment which makes it possible to boil out wax. By M. S. Sorley, Chicago.

"Macroscopic and Microscopic Dentoalveolar Pathology." Microscopic slides from sections through alveolar process with teeth in situ with case histories, post mortem findings of patients and ante and post mortem radiograms. By C. S. Suddarth, Chicago.

"Movable—Removable Bridge Construction," by Thomas P. Rose, Chicago.

"Fixed Bridge Attachments for Vital Teeth." Demonstrating with the aid of large models, a method of attachments as applied to vital teeth. By G. R. Churchill, Chicago; F. G. Conklin, Chicago; H. C. Lee, Chicago.

"Making Gold and Aluminum Castings with an Automatic, Electric, Pyrometer Machine." Demonstration of a unique method of employing a combination of gold and aluminum in partial denture construction—casting both metals into one mold. By F. E. Roach, Chicago.

"Combined Chair and Table Clinic with Patient."

A. Ful maxillary and mandibular continuous gum dentures, built by Rene Cournand of Paris, France.

B. Ful maxillary and mandibular dentures illustrating different arch forms with modifications in tooth arrangements for class one, two and three ridge relations.

C. Corrected case of maxillary prognathism with technic models of steps in treatment. Photographs of patient before and after treatment.

D. Application of continuous gum on partial dentures and bridgework.

E. A Porcelain Shell Crown.

F. Porcelain Jacket Crowns. By R. O. Schlosser and J. F. Christiansen, Chicago.

"Root-Canal Technic," by L. L. Davis, Chicago.

"Fixed Sterilization of Teeth Using Violet Blue Briliant Green," by E. L. Griffith, Freeport.

"Porcelain Dowel Crowns." Technic for anterior and posterior dowel crowns, using all porcelain teeth, obtaining accurate adaptation—Maximum strength and aesthetic effect—reducing to a minimum gingival irritation. By C. M. Smith, Peoria.

"Repalating Upper and Rebasing Lower Dentures." Practical demonstration of service to the patient using "Impression Compound" in full dentures built over soft irregular ridges; also technic for "Repalating the Upper" and the "Rebasing of Lower" dentures. By Virgil P. Perisho, Streator.

"Single Cast Removable Bridges for Vital Teeth," by Frank E. Roe, Streator.

"Gold Inlays and Fixed Bridge Attachments," by P. A. Pyper, Pontiac.

"Cavity Preparation and Construction of Cast Gold Inlays by the Indirect Method." This clinic will deal with complicated cavity preparation in an attempt to show the practicability of the use of the gold inlay for extremely large and difficult restorations. By Arthur L. Lee, Flanagan.

*Chicago Prosthetic Clinic Club*

W. H. Heermans, Director

"Full Denture Construction." Preliminary impressions; final impressions; mandibular registrations; freways; casts and mounting; arch forms; occlusion; mounting for regrind; vulcanization; rebasing; regrinding; adjustments. (To be given by ten clinicians.)

*Indiana*—(Expected to furnish eight clinicians.)

"Orthodontia Treatment with Ribbon Arch," by E. L. Mitchell, Indianapolis.

"Technic for Full Upper and Lower Using Deep Bite Posteriors." Normal Overbite, Prognathous and Orthognathous. By Walter E. Beyer, Indianapolis.

"Using a Flat Pin Facing as a Removable Facing in Crown and Bridgework." The flat pin designed to take the place of the round pin facing in soldered crown and bridge construction is still subjected to heat and difficult of repair which by this technic is elminated. By D. A. House, Indianapolis.

"Fixed Bridge and Denture Clinic." Upper and lower posterior sanitary fixed bridges with three-quarter abutments for vital teeth, as constructed by using the jaws as an articulator; showing distinct advantage of a swedged upper gold denture over a cast and swedged upper gold denture. By Robert H. Hopkins, Indianapolis.

"Partial Plates With Cast Clasps Using Modeling Compound Impressions," by J. B. Carr, Indianapolis.

"Indianapolis Dental Credit Bureau," by Robert White Blake, Indianapolis.

in the mold cavity when the gold is entering same by removing air resistance. By L. L. Poston, Council Bluffs.

"A Quick Casting Method." A partial boil-out and quick carbonizing casting process by which the mixing, setting and burning out may be done in less than fifteen minutes. By E. I. Rosheim, Roland.

"Public School Dentistry." A quick and easy method of filling, prolonging the life, and preventing decay of deciduous teeth and six-year molars in extreme cases of children difficult to control. By Grayson G. Garrison, Burlington.

"Cavity Preparations and Castings for Gold Inlay and Three-Quarter Crowns." Explanation of the importance of tooth form, cavity preparation, carving and casting to obtain successful inlays; also instruments used in operations. By H. J. Altfillisch, Dubuque.

*Kansas*—(Expected to furnish six clinicians.)

(Title to be furnished later), by D. T. Parkinson, Wichita.

(Title to be furnished later), by E. E. Welch, Topeka.

(Title to be furnished later), by G. A. Crise, Manhattan.

(Title to be furnished later), by E. J. Cutshaw, Phillipsburg.

(Title to be furnished later), by C. E. Tuttle, Wichita.

(Title to be furnished later), by H. M. McFarland, Kansas City.

*Kentucky*—(Expected to furnish five clinicians.)

"A Practical Demonstration of the Details, in the Investing and Casting of Full and Partial Dentures," by L. C. Burgard, Louisville.

"Some Applications of the Casting Process to Crown and Bridgework." Restoring broken porcelain crown with casting plus veneer of baked or synthetic porcelain. Cast crowns and pontics with porcelain veneer. Technic of the application of synthetic veneer. By Edward Hubbuch, Louisville.

"A Denture Prognoanalysis Chart." A means by which we may measure the success of a denture, locate troubles, apply remedies, and have a preliminary understanding of the patient. By Robert Gillis, Hammond. "Accurate Post Damming." Method of post damming while plate is being constructed to know exactly where and how much post damming is needed. By Charles A. Priest, Marion.

*Iowa*—(Expected to furnish nine clinicians.)

"Porcelain Jacket Crown," by Craig M. Work, Ottumwa.

"Fixed Bridgework with Particular Reference to Diagnosis and Ceramics and Construction of Broken Stress," by Donald E. Smith, Sioux City; J. A. Bliss, Sioux City; E. W. Howard, Des Moines.

"Surgical Pyorrhea." In advanced cases a portion of the gum tissue will be removed and the alveolar process curetted; in mild cases curetting of the process only. By W. J. Charters, Des Moines.

"Gold Foil Restoration with Special Attention to Toothform," by H. A. True, Des Moines.

"Facial Casts Before and After Orthodontia Treatments." Full plaster facial casts showing how the profile can be improved by correcting malocclusion. By R. W. Noland, Des Moines.

"A Gold Jacket Crown." A new technic for the construction of a cast crown, showing ease in construction and assuring a proper fit after casting. By Joseph B. Kennedy, Des Moines.

"Three-Quarter Crown, A Special Attachment and Gold Inlay Used in Fixed Crown and Bridgework." Models of preparations in wax and finished castings—methods used in preparation, etc., where and when used; finished bridges mounted on technic casts. By J. Frederick Walter, McGregor.

"A Method of Investing Inlay Models and the Elimination of Wax and Moisture." A method of safely removing the wax and providing for the escape of air

"A Definite Technic of Transfer of Bites to an Articulator," by I. H. Harrington, Louisville.

"Fixed Bridge Replacement with Positive Support which Allows for Normal Tooth Movement," by Raymond E. Grant, Louisville.

"A Demonstration of Cast Gold Denture Technic," by Garnett H. Smith, Louisville.

"Partial Dentures." A few diversified conditions indicating partial denture construction with and without compensation anchorage for some of the simpler forms of clasp attachments. By William Randall, Louisville.

*Louisville Inlay Unit*

"Practical Cavity Preparation," by George H. Means, Louisville.

"Wax Manipulation and Investing," by Robert L. Sprau, Louisville.

"Wax Dissipation and Casting," by W. E. Goepper, Louisville.

*Kentucky Local Anesthesia Unit*—"Conductive and Infiltration Anesthesia." Indications and Contra Indications; Special attention to the Anatomy of Parts; Armamentarium, Preparation of Patient; Preparation of Solution; Simple Technic for all Injections. By E. C. Hume, Louisville; A. P. Williams, Louisville; Frank B. Hower, Louisville; M. B. Guthrie, Lexington; James S. Dailey, Lexington.

*Louisiana*—(Expected to furnish three clinicians.)

*Mississippi*—"Cast Gold and Porcelain Restorations," by W. C. Dennis, Jackson; R. S. Neyland, Jackson; G. S. Handy, Natchez; E. D. Hood, Tupelo; J. N. C. Moffat, Clarksdale.

"A New and Novel Method of Thoroughly Cleaning the Mouth," by Dr. J. N. C. Moffat and three nurse assistants, Clarksdale.

*Maine*—(Expected to furnish —— clinicians.)

*Maryland*—(Expected to furnish three clinicians.)

"Wire Arch Adjustments with Heat." A method of making and measuring accurately the slight successive changes necessary to control and direct the spring tension of wire arches in their relation to moving teeth. By Norris C. Leonard, Baltimore.

*Massachusetts*—(Expected to furnish eleven clinicians.)

"Arch Predetermination."

1. Method of determining tooth substance in a given arch.

2. Method of surveying the malformed arch.

3. Method of using predetermined arch on cases of malocclusion to show required movement.

4. Case models, showing the practical application of the normal orthodontographic arch from the starting of case, through transition period to completion, and two years after all appliances have been removed. By William H. Gilpatric, Boston.

"Ceramics." Table clinic and exhibit of a variety of porcelain crowns, clasp removable bridges, and fixed porcelain bridge on a platinum frame of new design. By W. G. Bridge, Boston.

"Denture Prosthesis, Full and Partial" (Group Clinic), by W. H. Hoyt, West Somerville; F. W. Allen, Boston; R. Ruelberg, Brighton; A. Gahm, Brighton.

"Dental Orthopedics." Expansion of narrow temporary arches permitting the proper growth and eruption of the permanent teeth without orthodontic aid, giving natural function, beauty and efficiency. By Moses Joel Eisenberg, Roxbury.

"The Etiology, Diagnosis and Treatment of Early Periodontal Lesions." Illustrations and lantern slides of radiograms showing periodontal disintegrations from the ages of twelve to twenty-one, including an explanation of the proper correction of a traumatic occlusion. By Benjamin Tishler, Boston.

"Fundamentals in Technic of Partial Denture and Crown and Bridge Prosthesis." A demonstration of the various steps in crown and bridge, clasp and simple partial denture construction. By George Phillips, Boston.

*Michigan*—(Expected to furnish eleven clinicians.)

*Detroit Clinic Club*

Preventive Dentistry

"The Prevention of Diseases and Conditions Which May Result in the Destruction of the Hard Structures of the Teeth," by Ruth M. Conway, Detroit.

"The Prevention of Diseases and Conditions Which May Result in the Destruction of the Dental Pulp," by William F. Northrup, Detroit.

"The Prevention of Diseases and Conditions Which May Result in the Destruction of the Periodontal Tissues," by Clayton H. Gracey, Detroit.

"The Prevention of Diseases and Conditions Which May Result in Malocclusion," by Harry T. Wood, Detroit.

"Preventive Dentistry," by Russell W. Bunting, Detroit.

"The Value of Record Charts and Study Models to Preventive Dentistry," by George C. Bowles, Detroit.

"The Relation of Hygiene to Preventive Dentistry," by Grace Rogers Spalding, Detroit.

*Battle Creek Clinic Club*

"A Method of Constructing Abutment Pieces for Vital Teeth." Tooth Preparation. By B. F. Johnson, Battle Creek.

"Construction of Anterior Abutment Piece," by R. A. Roelofs, Battle Creek.

"Construction of Posterior Abutment Piece," by F. D. Loomis, Battle Creek.

"Finished Models," by C. S. Larned, Battle Creek.

*Detroit Clinic Club* (*Surgical Section*)

"Anesthesia, Block and Infiltration."

1. Sterilization, drugs and armementarium.

2. Showing technic by use of dry specimen.
3. Showing technic by use of wet specimen.
4. Showing technic by use of waxed specimen.
5. Showing technic by use of slides and moving pictures. By Don M. Graham, Detroit; W. A. Cook, Detroit; R. G. Hayward, Detroit; J. W. Kemper, Detroit; C. J. Hall, Detroit; Clare Straith, Detroit.

*Detroit Dental Clinic Club*

R. L. Girardot, Director

"Technic for Handling Pulpless Teeth." A complete course of instruction will be given in four progressive clinics as follows:

"Diagnosis and Radiography," by John H. Longe, Detroit; B. D. Welling, Detroit.

"Therapeutics," by Archibald C. Thompson, Detroit; Edward M. Slocomb, Detroit.

"Operative Technic, Equipment and Asepsis," by Earl C. Barkley, Detroit; Richard E. Patterson, Detroit.

"Filling the Root," by Harold S. Slocum, Detroit; Gerald F. Madison, Detroit.

*The Diack Clinic Club*

"The Importance of the Intangible Features of the Occlusal Surface." Presenting a technic for the restoration of the occlusal surface, consistent with a new conception of the function of mastication, as indicated by recent studies in comparative dental anatomy. By Alex Diack, Director, Detroit; D. C. Arner, Detroit; Louis Braun, Detroit; Phillip A. Callahan, Detroit; M. W. Frost, Detroit; H. W. Kohn, Detroit; M. H. Miars, Detroit; O. C. Williams, Detroit.

"Complete Oral Diagnosis." A complete oral diagnosis necessarily includes:

1. Knowledge of systemic conditions.
2. Careful clinical examination.
3. Correct radiographic examination.
4. Correlation of systemic, clinical and radiographic findings. By H. H. Jackson, Detroit; B. D. Welling, Detroit; Carleton Fox, Detroit; John H. Longe, Detroit; George W. Christiansen, Detroit.

*Minnesota*—(Expected to furnish eleven clinicians.)

"Exodontia—Post-Operative Complications." Hemorrhage—over-surgery. Treatment of infection and other post-operative conditions. By Henry B. Clark, St. Paul.

"Constructive and Destructive Pathological Processes in Teeth and Bone." Illustrated with lantern slides. By J. A. Heidbrink, Minneapolis.

"The Color Problem in Porcelain Restorations in the Mouth." The science of chromatics as it is related to the subject, the technic of color selection, the problem of properly interpreting color in our porcelain restorations. By William D. Vehe, Minneapolis.

"The Porcelain Veneer Crown." The principles of tooth preparation for the crown; impression taking; cast and die making; matrix making; building and completing the crown. By J. Raymond Gill, St. Paul.

"Practical Application of Dietetics for Children from the Dental Viewpoint." Demonstration of practical dietetic diagnosis in dental office; demonstration of limitations and factors to be considered in dietetic changes suggested by dentist. What is Safety First in advising dietary? By Philip R. Thomas, Minneapolis.

"Diseases of the Periodontium." A chart and model clinic giving types, causes, and methods of treatment, past and present, of gingivitis, dental periclasia and ulcerative stomatitis. By Harold J. Leonard, Minneapolis.

*Monson Research and Clinic Club*

"Fixed Reconstruction." Attempting to show that the solution of the problem of fixed reconstruction is largely dependent upon the occlusal balance obtained. Also showing a balanced occlusion guide (B.O.G.) by which balanced occlusion may be obtained in fixed reconstruction. By the aid of complete clinic material this demonstration will proceed from diagnosis of mounted casts to the completed case. By C. K. Bird, St. Paul.

"Removable Bridgework." A detailed demonstration of the steps involved in the restoration of occlusal balance by the use of removable bridgework; showing that success depends upon balanced occlusion more than any other one factor. By the use of specially constructed casts we shall attempt to determine the degree of malocclusion; the establishment of a new occlusal line; the amount of opening necessary; a new mesiodistal relationship of mandible to maxilla. Also we shall show operative procedures for restoring abutment teeth to arc. Finally the importance of tooth anatomy as applied to occlusion in the fullest sense. By O. DeForest Davis, Minneapolis.

"Full Denture." A complete demonstration with the aid of clinic material and a specially devised lantern by which the clinic is supplemented with slides showing the detailed steps on a patient. We shall attempt to demonstrate a simple impression technic; the importance of obtaining the same relationship of masticating units on the articulating instrument as in the patient; correct set up of teeth and a waxing up with the idea in mind of permitting free enunciation in speech; finally the significance of obtaining a balanced occlusion, not only on the instrument but that it shall be balanced in the mouth as well. By H. B. Washburn, St. Paul.

*Missouri*—(Expected to furnish nine clinicians.)

"Anchored Dentures." Spring relief, unilateral and bilateral cases using the cast clasp. Cast clasps in combination with inlays. Direct and indirect retention. By George B. Scott, St. Louis.

"Work of the St. Louis Study Club." Explaining the system of teaching followed in the different courses and presenting some of the technic as required by the different instructors. By Otto J. Fruth, St. Louis.

"Oral Radiography." Illustrating the necessity for thorough radiodontic examinations and discrimination in interpreting the evidence. By Clarence O. Simpson, St. Louis.

"Technical Phases of Inlay Construction." A demonstration of technical procedures to improve the efficiency of cast restorations. By Edward E. Haverstick, St. Louis.

"Removable Bridgework, Replacing Lost Teeth Distal to Cuspids." Unilateral and bilateral bridge, so constructed as not to bear strain on abutments. Practical cases will be shown that have been in service from eight months to three years. By Charles P. Grosby.

"Root-Canal Technic and Treatment of Pulpless Teeth," by W. A. Chamberlain, St. Louis. (Additional names to be supplied.)

"Instruments and Equipment with Demonstrated Technic, for Doing an Aseptic Root-Canal Operation." Infection is the greatest factor causing failure in root-canal work. Second to infection is chemical and instrument trauma. Our clinic is to demonstrate methods for preventing these. By Edouard M. Hall, Kansas City.

"Why Metal Bases Should be Swedged and How to Swedge Them." The inherent qualities of expansion and contraction in metals make swedging of full bases imperative as a complement to the casting process. Simplicity of technic renders the omission of swedging entirely inexcusable. By Dayton Dunbar Campbell, Kansas City.

*Montana*—(Expected to furnish two clinicians.)

*New York*—(Expected to furnish twenty clincians.)

"Orthodontia." Plan of Diagnosis. The orthodontic treatment of deformities of the jaws and any irregularity of the teeth. Illustrated with a movable mandible and models with apparatus. By V. H. Jackson, New York City.

"A Matrix for Amalgam Fillings, Making Possible the Correct Formation of Point of Contact." A matrix in two parts. The filling is built against the proximate tooth and the matrix, being separable, is removed from beneath the contact point. By John T. Hanks, New York City.

"Roentgen Interpretation of the Teeth, Jaws and Sinues," by Samuel M. Getzoff, New York City.

"A New Technic for the Taking of Dental Radiograms, Extra and Inter-Orally, by Predetermined Angulation." Demonstration on patients of new technic for taking extra, intraoral, and stereoscopic radiograms by predetermined angulation. Radiograms thus taken can be exactly duplicated after any length of time. By John F. Freund, New York City.

"Post-Operative Treatment of Mouth Wounds." System of post-operative treatment based on drainage which practically eliminates post-operative pain and infection. Discussion of the mouth uses of anilin dyes of the Flavine Series. By Arnott A. Moore, Buffalo.

*Buffalo Study Club of Dental Engineering*
Dr. H. E. Reynolds, Director

"Full Denture Technic, Complying with Individual Requirements of the Patient; Using the Adjustable Hanau Articulator." Occlusal rims, Central Occlusion, Face Bow, Bites, Adjustment of Articulator. By H. E. Reynold, Director, Buffalo; E. J. Farmer, Buffalo.

"Face Bow Technic and Its Importance," by H. E. Reynolds, Buffalo.

"Adjustment of Condylar Guidance in the Articulator, Condylar Guidance and Its Effect Upon Balanced Occlusion," by H. T. Gallager, Buffalo.

"Adjustment of Incisal Guidance in the Articulator. Overbite, Aesthetics, Phonetics. Incisal Guidance and Its Effect Upon Balanced Occlusion," by E. R. Melcher, Buffalo.

"Selection of Teeth; Cusp Height and Its Effect Upon Balanced Occlusion; Correction Technic," by George Snider, Buffalo.

*The Buffalo Prosthetic Clinic Club*
Dr. Walter F. Chappelle, Director

"Full Denture Technic, Valuable Aids to Better Denture Service." "Salesmanship and Instruction to Patients—the Use of Psychology Makes Satisfied Patients Anxious to Co-operate," by A. E. Wimmack, Buffalo.

"Ridge Relationship and the Use of a Face-Bow With an Arc and Adjustable Level Arm to Properly Place Models in the Articulator so that the Resulting Condyle Path Angles Will Approximate Natural Conditions," by S. A. Gibson, Buffalo.

"A Simple Method of Checking Up Central Occlusion—Developing Aesthetics—Wax Protrusion Bites," by A. J. McCarthy, Buffalo.

"Setting Up Posteriors for Co-ordination and Balanced Bite with a New Anatomical Adjustable Articulator," by W. F. Chappelle, Buffalo.

"Waxing, Flasking, Packing and Finishing, Stressing Many Short Cuts and Aids to Better Work. Regrinding for Minor Corrections," by L. A. Starks, Buffalo.

"A Simple Correction Technic for Locating and Correcting Errors of All Kinds," by Leon Algire, Buffalo.

*University of Buffalo Clinic Club*
Charles K. Buell, Director

"Complete Technic for the Construction of a Hand-Carved Porcelain Shell Crown—By the Direct Method." "Model and Drawing Exhibit with a Talk Upon the Advantages of This Crown Together with the Technic of Tooth Preparation," by Daniel H. Squire, Buffalo.

"The Technic of Matrix Making and the Readapting of the Matrix Tooth After Biscuit Firing," by Albert B. Cutler, Buffalo.

"Technic of the Application of the Porcelain to the Matrix, Especial Emphasis Being Given to the Blending of Colors and the Carving of Porcelain," by Joseph L. Cleveland, Buffalo.

"The Firing of Porcelain, Both the Biscuit and the

Final Firing, with the Removal of the Matrix and an Exhibit of Finished Crowns,'' by C. K. Buell, Buffalo.

*New Mexico*—(Expected to furnish two clinicians.)

''(Title to be furnished),'' by R. E. Coulter, Raton.

*New Hampshire*—(Expected to furnish two clinicians.)

*North Carolina*—(Expected to furnish three clinicians.)

''Technic on a Few Original and Practical Points in Dentistry,'' by Charles L. Alexander, Charlotte.

''Plaster Mask of Adult Tubercular Mouth Breather, Showing Results of Operation, Restoring Nasal Breathing,'' by J. N. Johnson, Goldsboro.

''A Method of Constructing a Cast Crown,'' by L. G. Coble, Greensboro.

*North Dakota*—(Expected to furnish three clinicians.)

''Gold Inlay,'' by L. I. Gilbert, Fargo.

''Tooth Form,'' by H. C. Cooper, Abercrombie.

''Gold Foil,'' by Fargo Study Club.

*New Jersey*—(Expected to furnish eight clinicians.)

''Etiology of a Type of Orthodontic Cases.'' Showing how many cases of malocclusion develop due to the lack of early observance or knowledge of conditions by the family dentist. By Charles S. Hardy, Summit.

*National Capital*—(Expected to furnish two clinicians.)

*Nebraska*—(Expected to furnish five clinicians.)

*Ohio*—Expected to furnish fifty clinicians.)

*Toledo Clinic Club*

''Gold Castings, Indirect Method.'' ''Cavity Preparation,'' by L. L. Barber and W. J. Dierks, Toledo.

''Impression and Bite,'' by W. S. Graves, Toledo.

''Making and Articulating Amalgam Models,'' by J. W. Travis, Toledo.

''Making Wax Pattern,'' by W. J. Hartshorn and R. H. Vollmayer, Toledo.

''Investing, Elimination of Wax, Casting,'' by R. K. Wood and W. H. Hisey, Toledo.

"Polishing, Cementing and Finished Work," by A. H. Breitenwischer, Toledo.

"Improved Amalgam Technic," by G. F. Bell, Conneaut; S. W. Brown, Ashtabula.

"A Combination of Three-Quarter Crown and Inlay Bridge Attachment with Tooth Form Dummy, Porcelain Cervical," by Frank C. Starr, Columbus.

"Wrong Claps in Removable Bridge and Partial Dentures," by M. E. Niswonger, Dayton.

"Chemistry and Physics of Alloys," by Mr. Selner, Cleveland.

"Fuller Lower Denture," by M. H. Stewart, Findlay.

"Full Dentures," by Cincinnati Clinic Club (ten men participating).

*Cleveland School Clinic Club*

"Amal Restoration, Deciduous Teeth," by Guy S. Lerch, Cleveland; W. M. Goodwin, Cleveland; F. E. Miller, Cleveland.

"Surgical Clinic" (Mt. Sinai Hospital), by M. B. Galvin, Cleveland.

"Crown and Bridge Technics," by F. G. Behmlander, Cleveland.

"Operative Technics," by E. W. Mullenkopf, Cleveland.

"Surgical Clinic" (City Hospital), by P. J. Aufderheide, Cleveland; M. L. Drake, Cleveland; Will Nichols, Cleveland.

*Oklahoma*—(Expected to furnish five clinicians.)

"A Method of Constructing a Soldered Base Porcelain Crown," by Wilbert J. Scruton, Oklahoma City.

"Original Technic for Lower Denture." Demonstrating on anatomical specimen, special fitting of tray and taking of impression, showing area fitted and fitted case. By W. T. Jacobs, Muskogee.

"Exodontia," by J. B. Jenkins, Oklahoma City.

"Root-Canal Technic." Reasons for combined use of sodium potassium and sulphuric acid in opening root-canals. Clinical demonstration of a complete technic, including cultural methods and electrolytic medication. By A. L. Walters, Tulsa.

"(Subject to be supplied)," by L. M. Doss, Oklahoma City.

"(Subject to be supplied)," by Charles Hess, Idabell.

*Oregon*—(Expected to furnish three clinicians.)

"Considerations in Treatment of Cases Requiring Pronounced Development," by E. M. Griffin, Portland.

*Pennsylvania*—(Expected to furnish seventeen clinicians.)

*The Dental Clinic Club of Philadelphia*

"Removable Restorations."

1. Cavity preparation for inlays, etc.
2. Making wax patterns and skeleton patterns.
3. Impressions, sectional methods.
4. Making different types of attachments.
5. Assembling. By W. J. Robinson, Director, Philadelphia; A. Kassab, Chester; Leon Halpern, Philadelphia; H. D. Shields, Philadelphia; J. H. Yearick, Philadelphia.

"Impressions of Edentulous Mouths Combining Correctible Plaster with Correctible Compound." With suitable casts and impressions the various steps necessary for securing full upper and lower impressions will be considered in detail. Both correctible plaster and correctible compound will be employed in the same case. By M. M. DeVan, Philadelphia.

"The Technic of Amalgam Work with Special Reference to the Use of Cement and the Matrix." Preparation of cavity for amalgam; Introduction and packing of amalgam; Trimming and carving to contour and accommodation of occlusion. By S. Blair Luckie, Chester.

"Fundamental Principles of Orthodontia for the Beginner." Analysis of the troubles which beset the beginner; Technic for construction of the appliances; Review of typical cases of malocclusion; Description of treatment. By Andrew Francis Jackson, Philadelphia.

"Pulp Conservation." A germicidal treatment of the infected dentin of deep-seated cavities, particularly of the six-year molars of children. Showing method of making medicament, and its application to infected cavity. By Andrew J. Seeler, Philadelphia.

"Exodontia Problems." Various methods of removing teeth as indicated by their shape, size, relation to adjacent structures and other determining factors. By Agnew Irwin, Philadelphia.

"Interesting Oral Surgical Cases." Lecture will cover the diagnosis and treatment of surgical conditions of the jaws. Cysts—osteomyelitis of mandible—impactions within the orbit—tumors, etc. Alveolar abscesses with septicemia. By James R. Cameron, Philadelphia.

"A Simplified Technic for Bridgework with Porcelain at Gum Contact." Using a facing ground to shape and polished by a process that renders a glaze in smoothness that can not be distinguished by magnifier from oven glaze. By John G. Logan, Portage.

"A Method of Cast Clasp Technic for Partial Restorations." Selection of cases, impression technic, making of casts and materials used, waxing of cases, investing. Alloys used and their treatment. Heat treatment of casting. Polishing and finishing of cases. By Albert S. Mulford, Philadelphia.

"Local Intraosseous Anesthesia." A simple and safe method of securing immediate and complete anesthesia of individual teeth for extraction, cavity preparation and pulp removal. By J. Wayne Martin, Uniontown.

"A Simplified Wax Pattern Technic." A direct method for simple and compound inlays, three-quarter crowns and incisal angle restorations. The patterns will be carved on a manikin supplied with ivory teeth. By E. Bruce Clark, Uniontown.

"A New Principle in the Removal of Fractured Roots." The removal of fractured roots is a delicate operation that requires delicate instruments handled in a delicate way—the principle is new and the application simple—the time to remove a fractured root is at the time of the original operation and the post-operation pain is directly in proportion to the amount of traumatic injury produced. By Fred D. Miller, Altoona.

"Waxing, Investing and Casting." The manipulation and working ranges of wax and investing materials—the properties and working ranges of casting gold. By Emory C. Thompson, Warren.

*Prosthetic Research Club of Pittsburgh*
J. H. R. McCampbell, Secretary

"Full Denture Technic—Monson Spherical Plane of Occlusion." A two table clinic, giving the different stages in the construction of a full denture case, using the Monson instrument. First table: Impressions, Models, Face Bow and establishing normal central occlusion. Second table: Mounting on instrument and setting up teeth.

*Rhode Island*—(Expected to furnish three clinicians.)

*Southern California*—(Expected to furnish eight clinicians.)

"A Full Upper Continuous Gum Denture One One-Thousandth Platinum Foil," by Charles O. McBean, Los Angeles.

"A Semi-direct Gold Inlay Technic," by Nye White Goodman, Los Angeles.

"The Principles Involved in the Treatment of Mutilated Natural Dentures, Particularly Where Bridgework is Necessary," by B. B. McCollum, Los Angeles.

*South Dakota*—(Expected to furnish three clinicians.)

"Fractures of the Mandible." Illustrated lecture with Slides of Actual Cases from Oral Surgery Service of Cook County Hospital. By Ralph H. Fouser, Chicago, Ill.

"Prosthesis," by Robert Jasman, Sioux Falls.

*South Carolina*—(Expected to furnish three clinicians.)

*Texas*—(Expected to furnish six clinicians.)

"Restorations in Cases of Pyorrheal Absorption with Baked Porcelain Roots, Tips and Extensions, Using Saddle-Back Teeth and Long Pin Facings." Saddle-back teeth are used by baking extension on gum end, providing glazed porcelain next to gum tissue and porcelain grinding surface, no gold showing. Pins on facing bent at forty-five degrees giving more strength of attachment and allowing replacement of baked roots in case of breakage. By W. O. Talbot, Ft. Worth.

"Appliances for Treating Fractures of the Maxillae and Mandible." Showing skull with various appliances adjusted for the reduction of fractures of both maxillae and mandible. By J. M. Murphy, Temple.

"Wrought Clasps." Round and one-half round wrought wire clasps for attachments on removable and bridges and partial dentures. By E. L. Knox, Dallas.

"Method of Making Pins for First Bicuspids (Upper)." The use of half round wire for the making of double pin for upper first bicuspids. By Robert L. Wheless, Dallas.

"Special Pyorrhea Instruments and How to Make Them—Polishing Tooth Roots with Steel Instruments in Pyorrhea Treatment." Describing methods of using special types of instruments and demonstrating how to shape, temper and sharpen them—practical demonstration on extracted teeth. By Julian Smith, Dallas.

"Wire Clasps and Wire Framework for Partial Plates." A simple and practical application of the wire clasp and frame work reducing bulk of vulcanite and getting stabilizing pressure not gotten from the gold lingual and palatal bar. By J. O. Hall, Waco.

*West Texas Dental Society*
M. R. Garrison, Chairman

"Group Clinic on Removable Bridgework and Partial Dentures." (Names of Clinicians to be furnished.)

*Tennessee*—(Expected to furnish three clinicians.)

"Orthodontia," by Oren A. Oliver, Nashville.

*Utah*—(Expected to furnish three clinicians.)

*Vermont*—(Expected to furnish two clinicians.)

*Virginia*—(Expected to furnish five clinicians.)

"Construction of Bilateral Gold Partial Dentures by the Indirect Method." Impression with modeling compound and plaster in sections; impression boxed; amalgam packed in teeth ready for stone cast; stone and amalgam model; plaster bite; plaster bite packed with amalgam; model Articulated and set up in wax; exhibits showing the removal of wax for investing; finished case. Six cases completed showing principles of breaking stress on clasped teeth—three upper and three lower. By M. B. Rudd, Richmond.

"Art in Dentistry." A presentation of the application of art in dentistry, its necessity and advantages; dealing particularly with the anatomy of individual teeth and their relative positions. By Herbert Cohn, Richmond.

*Wisconsin*—(Expected to furnish nine clinicians.)

"Spring Pressure in Orthodontics." Models with appliances showing the method by which these springs act. By A. C. Gifford, Oshkosh.

"Fractures of Mandible and Maxilla." Some fundamental factors in their reduction, importance of X-rays in diagnosis, simple appliances and their practical application. Clinic to be illustrated with X-rays, models and appliances. By Roy S. Hopkinson, Milwaukee.

"Types of Fixed and Removable Bridgework," by O. G. Krause, Milwaukee.

"Factors Concerned in the Inception of Periodontal Disturbances and Ideas as to the Prevention of Recurrence," by Elbert J. Weaver, Milwaukee.

"The Obturator, Its Construction and Application in Connection with Cleft Palate," by Carl B. Case, Milwaukee.

*Superior Clinic Club*

Archibald G. Fee, Director, Superior

*Washington*—(Expected to furnish six clinicians.)

*West Virginia*—(Expected to furnish three clinicians.)

"Porcelain Shoulder Crowns." Preparation; adapting foil on teeth to be crowned; packing and the fusing of porcelain; staining of finished crown when necessary. By L. E. Lawson, Williamson.

"Surgical Preparation of Soft Tissues to Receive Dentures." A presentation by means of models, in plaster, showing teeth in position before extraction. Operation on soft tissues, chiseling, curetting, filling, etc., of alveolar processes, removal of teeth—suturing flap after treatment. By Howard W. Burnett, Fairmont.

*Wyoming*—(Expected to furnish two clinicians.)

### Section on Research

"Experimental Results of Planting Infected Teeth in Animals." A demonstration of the effect of dental infection as expressed in tissue lesions. By Weston A. Price, Cleveland, Ohio.

### Mouth Hygiene Section

Clinics in this section to be given in the Oral Hygiene Exhibit, open during the entire meeting.

(a) Exhibit of Lantern Slides for use in talks to the public on Oral Hygiene.

(b) Catalogue of slides for use of profession in arranging lecture and selecting and obtaining slides.

This exhibit arranged by Oral Hygiene Committee of the Canadian Dental Association, outlines a scheme whereby the state and local societies and the individual dentists may work in close co-operation in the important matter of Oral Hygiene lectures and propaganda. By Wallace Secombe, Montreal, Canada.

PARTIAL DENTAL SECTION

1. Preparation to show that Spherical surfaces allow balanced relation in all positions.

2. Spherical bite method showing central occlusion and range of occlusion.

3. Preparation showing relation of curve of Spee to condyle path and the depth of cusp and overbite.

4. Preparation showing that central occlusion may be established at any point within the range of occlusion by proper arrangement of inclined planes. By J. W. Needles, Pueblo, Colorado.

1. Use of transfer, its significance.

2. Mounting casts.

3. Checking same.

4. Diagnosis, showing each step in "positioning tie" and "balanced occlusion guide" technic. By C. K. Bird and Group, St. Paul, Minn.

1. Presentation of an abutment designed to combine the practical principle of the cast abutment with the aesthetic principle of the Porcelain Jacket Crown.

2. The form of the cast abutment to give approximal attachment to the pontic, combining with it a form for the reception of a Porcelain Jacket Crown, concealing all display of gold.

3. Preparation of the tooth.

4. The making of the wax impression in a copper band.

5. Formation of the wax model embodying the detail form for the cast abutment.

6. Fitting the abutment also giving the proper alinement to the pontic attachment.

7. Completing the bridge and the application of the Porcelain Jacket Crown operation, adding to the fixed bridge method the highest esthetic principle. By J. E. Argue, Seattle, Washington.

*Removable Bridgework*

The Fundamentals Underlying its Application in Partial Restorations.

1. The radiographic survey of Alveolar Ridge.
2. The application of the principle of broken stress.
3. Saddle adaptation.
4. Esthetics, color, arrangement, etc.
5. The handling of malposed teeth.
6. Occlusion. By B. B. McCollum, Director, Los Angeles, Cal.; D. W. McLean, Los Angeles; H. B. Holcomb, Los Angeles; G. M. Hollenback, Los Angeles.

*Clinic Outline*

Demonstration by a group of four or five men showing all of the steps involved in the diagnosis and reconstruction of a case of molocclusion resulting from loss of teeth. The above demonstration will include the technic of actual bridge construction, as well as showing finished cases restored to full occlusion.

Preparations on the various types of teeth for bridge abutments, preserving vitality. The application of dental anatomy as applied to the various preparations.

1. Preparatoins on various types of centrals and laterals. (A) Ovoid. (B) Square.
2. Preparations on cuspids. (A) Upper. (B) Lower.
3. Preparations on bell-shaped teeth. (A) Three-quarter veneer on upper and lower biscusips and molars. (B) Inlay Preparations.
4. Full crown veneers. (A) Lower biscuspids. (B) Upper and lower molars. By T. W. Maves, Minneapolis, Minn.

*Fixed Bridgework*

Problems and Important factors involved in manipulation of wax, investing process, carbonization and elimination of wax in the burning-out process, casting and casting gold.

To include:

1. Properties of wax.
2. Working ranges of wax.

3. Location of wax.
4. Getting the wax pattern.
5. Annealing the wax.
6. Removal of wax pattern.
7. Coating of wax.

Investment:

1. Properties of investment.
2. Working ranges of investment.
3. Proportions of investment and water.
4. Spatulation of investment.
5. Time to allow for crystalization.
6. Heating of investment.
7. Properties of casting gold.
8. Heating of gold.
9. A few fundamentals pertaining to Metallurgy. By Emory C. Thompson, Warren, Pa.

### Operative Dentistry Section

### *Gold Inlay Technic*

A. Finishing of cavity preparation. Cavity protection. Waxes, wax pattern and making and finishing.

B. Contour and contact.

C. Investment, Spatulation, application and setting.

D. Dissipation of wax, rapid and slow method. Temperature range.

E. Blowpipes and casting; oxygen gas; oxygen and hydrogen; hydrogen and air; hydrogen, gas and air.

F. Golds.

G. Finishing.

H. Setting. By V. T. Nylander and Assistants, Chicago, Ill.

